# How older people as pedestrians perceive the outdoor environment – methodological issues derived from studies in two European countries

**DOI:** 10.1017/S0144686X17000666

**Published:** 2017-07-31

**Authors:** HANNA WENNBERG, JUDITH PHILLIPS, AGNETA STÅHL

**Affiliations:** * Trivector Traffic AB, Lund, Sweden.; † Faculty of Social Sciences, Stirling University, UK.; ‡ Department of Technology and Society, Lund University, Sweden.

**Keywords:** walking, older people, urban design, mixed-method research, cross-national comparison, participatory research

## Abstract

This paper has re-analysed and compared data between three studies conducted in the United Kingdom and in Sweden (the OPUS ‘Older People's Use of Unfamiliar Space’ study in the United Kingdom and the Swedish studies ‘Let's Go for a Walk’ and ‘Walking in Old Age’) to provide a comprehensive account of the issues facing older people in the outdoor environment. All three studies draw on the ‘fit’ between the person and their environment as a guiding conceptual base – capturing the dynamics of the relationship between older people's personal needs and their wider environmental context. This common conceptual base allowed us to test theory against practice, and to explore the utility of this concept across different geographical contexts. Participatory research was also applied, highlighting the importance of the voice of older people and involving older people in research. The studies also used a mixed-method approach involving both quantitative and qualitative methods. The paper highlights that although not generalisable, you can compare cross-locales and cross-nationally using different methodology; it investigates the challenges of cross-national comparative analysis and draws on findings from the three studies to illustrate the different challenges and solutions and finally looks at lessons that are transferable.

## Introduction

The outdoor environment is an increasingly important area of study within gerontology. Likewise, the various needs and preferences of older people are crucial knowledge within the field of transportation and urban design. Research on older people's perception of the outdoor environment contributes to the development of age-friendly communities and cities as well as to the design of public spaces promoting the mobility and wellbeing of older people (Ståhl, Horstmann and Iwarsson 2013; Sugiyama and Ward Thompson 2007; Wennberg, Hydén and Ståhl 2010). The importance of green areas such as gardens and parks, wilderness areas and rural vistas have increasingly been recognised as important for people's health and wellbeing (Ward Thompson, Roe and Aspinall 2013). This is also true of green urban areas (Ward Thompson *et al.* 2012), although in this paper we address the built urban environment. Adapting outdoor environments to the needs and preferences of older people is also a critical safety factor in terms of preventing falls (Li *et al.* 2006; Ståhl and Berntman 2007).

Exchanging knowledge and understanding of how the outdoor environment can impact on older people's mobility and wellbeing and how older people can take part in shaping of their environment is crucial. The search for global solutions to the needs of our ageing populations in relation to outdoor environment have led to a number of European cross-national studies on older people and their environments, *e.g.* ENABLEAGE, MOBILATE, SIZE and AENEAS (Iwarsson *et al.* 2004; Mollenkopf *et al.* 2004*a*; Risser, Haindl and Ståhl 2010; www.aeneas-project.eu). Such studies have followed similar methodologies in each participating country, yet very little is written reflecting the success (or pitfalls) of the methods used in comparison to other studies on the same themes, unless they are part of a retrospective systematic review (Annear *et al.* 2013; Rosso, Auchincloss and Michael 2011).

In contrast to common methodology, this paper takes a different approach, exploring how three country-specific studies (the OPUS ‘Older People's Use of Unfamiliar Space’ study in the United Kingdom (UK) as well as the Swedish studies ‘Let's Go for a Walk’ and ‘Walking in Old Age’) can be re-analysed and compared to provide a comprehensive account of the issues facing older people in their outdoor environments. One of the primary aims of this paper is to explore how different research questions and methods of researching older people in the context of their local outdoor environment can highlight the difficulties, challenges and advantages of using different methodologies to compare findings from three studies in two country contexts. To place the studies in a wider context, the paper draws on the literature on outdoor environments and issues for older pedestrians, and the specific physical and social contexts in which the studies were conducted. Secondly, the paper outlines the importance of cross-national, mixed-method and participatory research. Third, it outlines the methodologies adopted by the studies and the methods used. Fourth, drawing on the main findings from the above three studies, it discusses advantages and success factors of a comparative approach as well as on the considerations when comparing data and drawing conclusions from the three studies. Finally, the paper provides methodological recommendations for future studies on older people's mobility as pedestrians as well as implications for planning.

## Previous research on outdoor environments for older pedestrians

Walking is an important transport mode for older people as well as an essential way of getting out and about, for exercise, recreation and joy (Iwarsson, Ståhl and Löfqvist 2013). Even though car driving is the most common travel mode amongst older people, transportation as a pedestrian or in public and special transport services becomes more important as people age (Rosenbloom and Herbel 2009; Stjernborg, Melin Emilsson and Ståhl 2014; Wennberg, Ståhl and Hydén 2009*a*; Whelan *et al*. 2006). In fact, 30–50 per cent of older people's journeys are made on foot in many European countries (Organisation for Economic Co-operation and Development 2001). Providing transportation options for non-drivers in the community, such as walkable neighbourhoods and user-friendly public transport, are therefore preconditions for many people to stay mobile and independent in old age (Michael, Green and Farquhar 2006; Mollenkopf *et al*. 2004*b*; Stjernborg, Melin Emilsson and Ståhl 2014).

Living in a neighbourhood with good community facilities and services (including transport) contributes to quality of life in old age (Banister and Bowling 2004). The design and maintenance of the outdoor environment facilitate people's ability to get out and about (Mollenkopf *et al.* 2004*b*; Risser, Haindl and Ståhl 2010). In particular, effective access to local shopping and services, attractive outdoor environments, the possibility to rest during a walk, good pedestrian facilities and access to public transport contribute to an independent active lifestyle in old age. In addition, design of pavements, seating and smooth pavements, walkways and other pedestrian facilities can support older people's independence and increase social interaction and community engagement (Hallgrimsdottir, Svensson and Ståhl 2015; Newton *et al.* 2010).

Previous research has emphasised various environmental barriers and fears when walking in urban areas. Barriers to older people's mobility are connected to traffic and infrastructure characteristics and older people often point out the importance of enforcing vehicle speeds as well as the design of barrier-free pedestrian environments and public transport. There are several barriers to good access in outdoor environments due to poor design and maintenance of pedestrian facilities. Such barriers included narrow pavements, poor crossing facilities, high kerbs, uneven or slippery surfaces, stairs without handrails, lack of benches, poor lighting, *etc*. (Carlsson 2004; I'DGO n.d.; Ståhl *et al.* 2008; Wennberg, Ståhl and Hydén 2009*a*).

Even though barrier-free design of pedestrian facilities is a prerequisite for the possibility for many older people to use the facilities at all, increased sense of safety and security is also essential in order to improve mobility, including both fear related to traffic and to crime (Wennberg, Hydén and Ståhl 2010). Risser, Haindl and Ståhl (2010) show that older people often point out the importance of perceived safety and security while experts tend to focus more on technical solutions (infrastructure, low floor vehicles). The differences in older people's and experts’ opinions are also examined by Ståhl *et al.* (2008), showing that older people often expressed a request for minor and not so costly measures (more benches, lowering kerbs, *etc*.) while experts expected more far-reaching high-cost proposals. Previous research illustrates the importance of involving older people themselves in research and planning in order to get a complete insight into the issues of older pedestrians (Carter and Beresford 2000; Phillips and Ray 2004; Ross *et al*. 2005).

The relationship between older people's needs and the environment in which they live and the potential mismatch between them is captured in Person–Environment fit, a theoretical approach developed by Lawton and Nahemow (1973). From their ecological theory of ageing, they propose that the lack of fit between the person (with different physical, sensory and cognitive competencies) and their environment (*e.g.* home, community, transport) can result in lower behavioural functioning and wellbeing. Person–Environment–Activity fit (Iwarsson and Ståhl 2003), a more recent addition developed out of occupational therapy theory, emphasises the fact that older people can shape and adapt their environment to suit their needs. The concept was used in all three studies to understand the impact of different environments and their walkability on older people, the residential satisfaction of older people and the extent to which older people could change their environment to suit their diverse needs.

## The importance of cross-national research

Similarities and differences between country-specific studies, especially between Scandinavian countries and the UK and between European countries and the United States of America (USA), are often highlighted through a literature review produced to contextualise the research issue. Researchers engaged in empirical cross-national research primarily use the same research tools in order to produce a systematic comparative outcome across spatial and cultural boundaries (Hantrais 2008). An explicit comparative frame of reference is developed and the data are interpreted within that framework.

Cross-national research can be useful for understanding one's own culture as well as highlighting diversity and providing transferable knowledge and practice between different settings (Denzin 1970; Hantrais 2008). Conducting cross-national research and gaining the benefits, however, does not necessarily need systematic use of the same methodological tools. Theoretical comparisons and theory building can come through comparative analyses of diverse data-sets. As inter- and multi-disciplinarity are encouraged and resource constraints of time and money vary between countries, it then becomes even more difficult to reach methodological equivalence in all countries under study which underlines the necessity of seeking further ways of comparative analysis.

There are inherent difficulties, however, in harmonisation of data and analysis when different methodological tools are used; similarly, it is difficult to make comparisons across different socio-cultural settings. One way to counteract this is to blend an etic approach (shared theoretical concepts) with an emic approach (participants’ words and interpretations). Although guided by pre-existing theoretical frameworks, participants’ words are important in interpreting and explaining cultural differences. The use of such an approach (as taken by the comparisons between the UK and Sweden in the paper) enables comparisons to be made and can allow researchers to develop broader cross-cultural themes and concepts (Olive 2014). One of the ways in which this blended approach can be strengthened is to undertake participatory research with participants as co-analysts. Consequently, when these two approaches are combined the ‘richest’ view of a culture or society can be understood. On its own an emic approach would struggle with applying overarching values to a single culture. The etic approach is helpful in enabling researchers to see more than one aspect of one culture and in applying observations to cultures around the world.

A ‘country effect’ is also highlighted by Berthoud and Iacovou (2002) who discuss in their paper on ‘Diverse Europe’ an alternative approach to cross-national comparison under the labels of micro-qualitative, micro-quantitative (detailed analysis of household data covering a range of countries) and macro-quantitative (comparison based on aggregated statistics). Under a micro-qualitative approach large quantities of both statistical and descriptive information are collected about each country to a harmonised agenda, but without a common data source the advantage of which lies in the detailed understanding of the processes at work in that country (Berthoud and Iacovou 2002). This latter method is of relevance to this paper, drawing on a similar approach.

Further difficulties in cross-national research are highlighted by Jürges (2007) who draws attention to the fact that respondents from different countries may have different response categories which have different connotations, particularly if they are self-reporting. Using the Survey of Health, Ageing and Retirement in Europe (SHARE) data as an example, he looks at self-reported health across countries and concludes that differences across countries are only party reflected by differences in true health. The remaining differences can be attributed to differences in reporting styles. To overcome some of the difficulties of interpreting cross-national data, the use of vignettes has been used to enhance the validity and cross-cultural comparability of measurements in survey research (King and Wand 2007).

## Methodologies adopted and methods used

To explore how research questions, methods and findings in the field of older pedestrians’ perception of the outdoor environment can be compared and applied given different contexts, this paper draws on three studies: OPUS, ‘Let's Go for a Walk’ and ‘Walking in Old Age’. The OPUS study is a cross-sectional study conducted in UK, while ‘Let's Go for a Walk’ and ‘Walking in Old Age’ are before and after studies conducted in Sweden involving interventions in the local outdoor environment. The three studies used a mixed-method approach involving both quantitative and qualitative methods. Tables 1 and 2 give an overview of the aims and methods used in the studies.

**Table 1.** Overview of the studies

Study	Aim	Design	Period	Country	Main publications
‘Older People's Use of Unfamiliar Space’ (OPUS)	Explore older people's use of unfamiliar environments. One of the research questions concerned factors that made environments worrisome for older people	Cross-sectional	2008–2011	United Kingdom	Phillips, Walford and Hockey (2011), Walford *et al.* (2011), Lewis and Phillips (2011), Hockey, Phillips and Walford (2013)
‘Let's Go for a Walk’	Investigate older people's perception of environmental measures taken in their residential area and changes in perceived difficulty as pedestrians and self-reported outdoor activity	Before–after	2002–2006	Sweden	Ståhl *et al.* (2008), Ståhl, Horstmann and Iwarsson (2013)
‘Walking in Old Age’	Examine older people's needs and perceptions as pedestrians and how measures to achieve year-round barrier-free outdoor environments impact older people's mobility and perceived safety	Before–after	2006–2008	Sweden	Wennberg, Ståhl and Hydén (2009*a*), Wennberg, Hydén and Ståhl (2010)

**Table 2.** Overview of methods used in the three studies

	OPUS (concurrent nested)	‘Let's Go for a Walk’ (exploratory and explanatory approach/transformative)	‘Walking in Old Age’ (exploratory and explanatory approach/transformative)
Quantitative methods	Questionnaire pre-cave (N = 44) • demographic information • cognitive functioning • self-perceived health • social networks • perception of residential neighbourhoods • navigational awareness • experiences Street audits (SWEAT-R + UDQ)	Postal questionnaire before (N = 347) and after (N = 195) • demographic information • travel habits • away from home activities • residential situations • access and safety problems • health and functional limitations	Postal questionnaire before (N = 356) and after (N = 244) • demographic information • usability and satisfaction • mobility and safety • functional limitations and use of mobility devices
Qualitative methods	Interviews and narratives in the cave Narratives from walk around town (N = 10) Focus groups with • older people • planners	Before • Participant observations^1^ (N = 6) • Inventory^2^ • Research circles (N = 16) • older people • planners and other experts After • Participant observations^a^ (N = 11) • Inventory^2^ • Focus group interview with older people (N = 5)	Before • Focus group interviews with older people (N = 9) • Participant observations^1^ (N = 4) After • Focus group interviews with older people (N = 10) • Participant observations^1^ (N = 3) • Interviews with municipal employees (N = 4)

*Notes*: OPUS: ‘Older People's Use of Unfamiliar Space’. SWEAT-R: Senior Walking Environment Assessment Tool – Revised. UDQ: Urban Design Quality. 1. Observed walk using critical incident technique (Flanagan 1954; Jensen, Iwarsson and Ståhl 2002). 2. Objective mapping of environment barriers using a standardised inventory protocol (Iwarsson and Slaug 2001, 2010; Steinfeld *et al.* 1979).

The OPUS study was based in two towns in the UK, Swansea and Colchester. Swansea is a university coastal city in south Wales, the second largest in Wales with a population of 242,000, 19 per cent of whom are over 65. Colchester in north Essex is the oldest town in Britain with a population of 180,000 of whom 17 per cent are over the age of 65. The Swedish studies were conducted in Hässleholm and Kristianstad, both medium-sized cities in the south of Sweden. Hässleholm has a population of 19,000 (23% are over 65) and Kristianstad 30,000 (20% are over 65).

### Method description

#### UK study

To explore issues of how older people (over 60) perceive and use unfamiliar space and what worries them about unfamiliar environments, a mixed-method approach was adopted in the OPUS study. First, quantitative and qualitative data were collected through a questionnaire and interviews conducted with respondents in a ‘reality cave’ where two-dimensional images and routes in familiar (Swansea) and unfamiliar (Colchester) towns were displayed; second, data were collected through street audits; and third, there was a site visit by respondents in the research to an unfamiliar town centre, where they followed the town route in real time and met with local planners and older residents in a focus group.

#### Swedish studies

The ‘Let's Go for a Walk’ and ‘Walking in Old Age’ studies also applied a mixed-method approach involving quantitative and qualitative research methods and emphasising involvement of older people (65+). Both are also before–after studies in which measures to improve accessibility and safety in the outdoor environment were implemented and evaluated.

To obtain different but complementary data on the same topic, the ‘Let's Go for a Walk’ study adopted a *triangulation design*, rendering comparison of findings as well as validation of results possible. The intent by using this kind of design was to bring together the different strengths and non-overlapping weaknesses of quantitative methods with those of qualitative methods (Patton 2002). The methods involved postal questionnaires, participant observations, objective mapping of environmental barriers, research circles (before intervention) and focus groups interviews (after intervention) (*see* Table 2). Further description of the methods used is found in Ståhl *et al.* (2008) and Ståhl, Horstmann and Iwarsson (2013). In the ‘Walking in Old Age’ study, focus group interviews and participant observations with older people were conducted in order to identify relevant usability factors concerning outdoor environments. These factors were then used in a postal questionnaire sent out on two occasions (before and after intervention in the study district) in order to examine the importance of the factors and how satisfied the respondents were with these factors. Such an *exploratory mixed-method approach*, with qualitative findings helping in developing and informing the quantitative method, is useful in terms of pre-screening potential respondents and their perception as well as other local preconditions concerning the study district and in the end formulating relevant questions for the questionnaire (Creswell and Plano Clark 2007). The questionnaire also examined mobility and perceived safety before and after interventions. For the interpretation of the results from the questionnaires, focus group interviews and participant observations were also conducted after intervention, *i.e.* an *explanatory mixed-method approach* was also applied. Further description of this procedure is found in Wennberg, Ståhl and Hydén (2009*a*) and Wennberg, Hydén and Ståhl (2010).

### Data collection

#### UK study

Prior to the cave exercise, respondents were asked to complete a *questionnaire* detailing demographic information and cognitive functioning (Cognitive Abilities Screening Instrument). The questionnaire also covered perceptions of their residential neighbourhood, social networks and navigational awareness. Each section consisted of questions scored on a five-point psychometric Likert scale. The residential neighbourhood section consisted of 35 variables and included aspects such as neighbourhood services, crime, traffic and aesthetics. The section on social networks consisted of 12 questions relating to relationships with others, including frequency of contacts with friends/relatives, as well as emotional and practical support within social circles. The navigational awareness section included measures on sense of direction (Santa Barbara-SBSOD), way finding (the Way-finding Strategy Scale), spatial anxiety in an unfamiliar area (Spatial Anxiety Scale) and barriers (Neighbourhood Environment Walkability Scale – Abbreviated). We were therefore able to examine matters such as strategies used in way finding and feelings of anxiety about unfamiliar spaces. The pre-cave survey included questions about visits to familiar and unfamiliar towns, dealt with the frequency of visits and modes of transport used, open-ended themes about why they visit, how they prepare, what they do, how they navigate and avoid getting ‘lost’, the usefulness of signage and presence of obstacles, situations and areas to be avoided, as well as general impressions and experiences of familiar areas. This paper draws on a selection of such methods to illustrate the issues of comparability.

In the virtual ‘reality cave’ respondents were shown images of familiar (their home area) and unfamiliar town centres and asked to comment on both still images and a 30-minute walk around an unfamiliar town. Seated next to the researcher, they were asked to remark on specific items during the journey, for example, the use of signage, confusing and helpful cues, colour, lighting, their confidence and the general impression of the route. Older people were asked to give a detailed narrative in relation to their reactions to and perceptions of unfamiliar spaces, as they journeyed through a route chosen by planners.

Respondents were asked for their comments and impressions of the predetermined route. The narratives were recorded, transcribed and thematically analysed drawing using the themes in the Urban Design Quality (UDQ) framework as detailed below.

Respondents were then engaged in street audits in their ‘home’ area; this was later repeated in the ‘unfamiliar’ area. The fieldwork in both familiar and unfamiliar areas included the Senior Walking Environment Assessment Tool – Revised (SWEAT-R; Michael, Green and Farquhar 2006) and UDQ index (Ewing *et al.* 2006). The first of these has been developed as a quantitative measure of the ‘walkability’ of urban environments, recording information about such physical characteristics as pavement width, kerb height and the presence of controlled crossing facilities. The second captures information about the quality of urban spaces, including such items as the range of building uses, the presence of amenity areas and planting. Together these provided a quasi-objective assessment of the condition, ambience and aesthetics of the urban environment along the route screened in the reality cave.

In order to capture the voices of older people and to get engagement with planners, ten respondents were later taken to the location of the unfamiliar area (Colchester in the East of England) to undertake a further ‘walk around town’ with researchers and older people from the town itself, enabling comparisons to be made between the cave environment and reality. All ten respondents were ambulatory and in good self-reported health. Here they followed the route projected in the cave and made assessments of their urban milieu using the SWEAT-R and UDQ. Additional qualitative data were collected through respondents recording their experiences in notes and through discussions with a group of local residents (ten) and planners. Consequently, the notes and focus group discussions were recorded and thematically analysed in the same way as the cave narratives, and SWEAT-R and UDQ measures were analysed, adding a subjective element, *i.e.* older people's voices, to the objective measures of the built environment. The cave experiment and survey were simultaneous with the ‘walk around town’ coming eight months after the cave experiment.

The focus groups with residents and the planners took the form of three meetings. One before and another after the walk around town (which included both residents and respondents), and a further meeting between our respondents and the planners of the town to discuss barriers and issues to improve the layout and accessibility of the town, as well as share the findings.

Further detail of the methods used can be found in Phillips *et al.* (2013), Phillips, Walford and Hockey (2011) and Walford *et al.* (2011).

#### Swedish studies

In both Swedish studies, a study-specific *postal questionnaire* was sent out before intervention, and a corresponding version of the questionnaire was sent out after interventions which were extended with questions concerning the respondent's perception of the different measures that were implemented in their local outdoor environment.

The questionnaires were designed to chart the travel habits and use of different transport modes, frequency of outdoor activity and residential situations of older people living in the study district, and to identify accessibility and safety problems in the outdoor environment, specifically along pedestrian walkways. The questionnaire also included questions examining how often outdoor mobility was avoided and reasons for such avoidance. In the questionnaire in the ‘Walking in Old Age’ study, one of the questions consisted of 27 usability factors identified in the qualitative before-studies. Here, the respondents were asked to state how important each one of the usability factors were on a five-point rating scale as well as how satisfied they were with the factors.

In order to engage the respondents more actively than is commonly the case in postal questionnaires and to focus their attention on the actual situation in their local outdoor environment, they were first of all asked to specify the most important destinations for their travels. In this context, they were asked to give concrete descriptions of which specific areas/passages they perceived as being inaccessible and/or unsafe, including identification of exact locations for their perceived problems along common itineraries. Another way to nurture active respondent involvement in both studies was to ask them for their proposals for eliminating the problems they reported.

Both studies also examined participant characteristics, such as age, sex, self-reported functional limitations, use of mobility devices and access to car or special transport services. Based on the items of the personal component of the Housing Enabler concept (Iwarsson and Slaug 2001, 2010), functional limitations and use of mobility devices were scored dichotomously (yes/no).

The postal questionnaire in the ‘Let's Go for a Walk’ study was comprised of 47 structured and five open-ended questions. The before-questionnaire was distributed in spring 2002 and the after-questionnaire in autumn 2006. In ‘Walking in Old Age’, the questionnaire consisted of 36 questions. The before-questionnaires were distributed by mail in May 2006 and the after-questionnaires in September 2007.

Both Swedish studies applied *participant observations* before and after interventions. The participant observations aimed to register environmental barriers and risk factors along the pedestrian walkways to important destinations close to the study district, as selected by each participant. The data collection methodology applied reflected subjective as well as objective perspectives of older people's interaction with the physical environment. Following the procedure in Jensen, Iwarsson and Ståhl (2002), the participant walked, accompanied by a researcher, to the selected destination and back again. During the walk, the participant reported what they experienced as problems in the environment while applying the critical incident technique (Flanagan 1954). The researcher also observed and registered the problems encountered by the participant. The data were recorded using a study-specific format, categorising different types of critical incidents according to pre-defined definitions (Jensen, Iwarsson and Ståhl 2002).

A short time after the participant observations were conducted, another researcher independently walked the same route as the respondents in order to register environmental barriers and risk factors in the outdoor environment from an objective perspective. This *objective mapping of environmental barriers* implies an objective evaluation of the pedestrian walkways in accordance with the methodology developed by Iwarsson and Slaug (2001) based on the Enabler Concept (Steinfeld *et al.* 1979) and further elaborated in Iwarsson and Slaug (2010). Assessments and measurements concerned design features such as the width of walkways, slopes, unevenness in the walkway surfaces, and number of and distances between places to sit down. Objective mapping of environmental barriers was done before and after the interventions.

In order to help in interpreting the results from the quantitative methods in the two Swedish studies, *focus group interviews* were also conducted after the interventions. The focus group interviews began with a general discussion about the usability of outdoor environments within the study district and as time went on the discussion was led towards the participants’ perceptions of the implemented measures. At this moment, the results from the questionnaires were also presented.

For the intervention in the ‘Let's Go for a Walk’ study, implemented measures were selected on the basis of *research circles* with older people, planners, stake-holders, property owners and decision-makers under the leadership of researchers as defined by, for example, Härnsten (1994). The research circles aimed to bring all parties with an interest in the issue together to create a programme for concrete improvements in the outdoor environment within the study district. The group met on five occasions to discuss problems and solutions. The results from postal questionnaires and participant observations were presented by the researchers and synthesised with the results emerging during the research circle discussions. A participant feedback check was also conducted at the end of each research circle meeting in which the researchers summarised the main findings back to the respondents and checked if their findings were an accurate reflection of their experiences (Creswell and Plano Clark 2007).

### Sample description

Table 3 shows the characteristics of the respondents in the quantitative parts of the three studies in terms of age, gender, household structure, functional limitations, perceived health, use of mobility devices, ability to walk certain distances and to carry out daily activities, frequency of walking and transport options.

**Table 3.** Characteristics of the respondents

	OPUS	‘Let's Go for a Walk’	‘Walking in Old Age’
N^1^	44	347	356
Mean age (years)	70.75	76.15	77.51
Age range (years)	59–84	65–99	65–99
	*Frequencies (%)*		
Age:			
65–79		228 (66.3)	202 (58.0)
80+		116 (33.7)	146 (42.0)
Gender:		
Women	29 (60.4)	215 (62.0)	222 (63.2)
Men	19 (39.6)	135 (38.0)	129 (36.8)
Household structure:		
Married/co-habitation	29 (60.4)	192 (55.8)	164 (46.7)
Unmarried	1 (2.1)	22 (6.4)	18 (5.1)
Divorced	12 (25)	34 (9.9)	37 (10.5)
Widow/widower	6 (12.5)	96 (27.9)	132 (37.6)
Couple household	29 (60.5)	192 (55.8)	164 (46.7)
Single household	17 (35.4)	152 (44.2)	187 (53.2)
Functional limitations:			
Movement	–	120 (51.7)	133 (37.4)
Perception/cognition	–	36 (15.5)	40 (11.2)
Movement/perception/cognition	–	62 (26.7)	98 (27.5)
No functional limitation	–	14 (6.0)	85 (23.9)
Perceived health:			
Mean score	–	5.02	4.81
1. Very poor	–	3 (0.9)	11 (3.2)
2.	–	8 (2.4)	24 (7.0)
3.	–	23 (6.8)	32 (9.3)
4.	–	79 (23.4)	65 (18.9)
5.	–	103 (30.6)	79 (23.0)
6.	–	74 (22.0)	85 (24.7)
7. Excellent, could not be better	–	47 (13.9)	48 (14.0)
Sometimes satisfied	5 (10.4)		
Quite often satisfied	15 (31.3)		
Always satisfied	28 (58.3)		
Mobility aids:			
Stick/crutch	–	33 (9.5)	51 (14.3)
Rolator	–	53 (15.3)	71 (19.9)
Wheelchair	–	13 (3.8)	22 (6.2)
No mobility aid	–	203 (70.0)	232 (70.3)
Ability to walk at least 200 metres:			
With support	–	42 (64.5)	–
Without support	–	259 (81.7)	265 (79.3)
Ability to walk at least 1 kilometre:			
Never	1 (2.1)	–	–
Very rarely	1 (2.1)	–	–
Less than once a week	3 (6.3)	–	–
Once or twice a week	26 (54.2)	–	–
Every weekday	5 (10.4)	–	–
Every day	12 (25.0)	–	–
Ability to carry out daily activities:			
Never	1 (2.1)	–	–
Sometimes	4 (8.3)	–	–
Quite often	8 (16.7)	–	–
Always	35 (72.9)	–	–
Frequency of walking:			
Every day	34 (70.8)	200 (59.5)	110 (31.8)
Several times a week	12 (25.0)	85 (25.3)	100 (28.9)
Once a week	1 (2.1)	26 (7.8)	84 (24.3)
Once a month	–	8 (2.4)	19 (5.5)
More seldom	1 (2.1)	17 (5.1)	33 (9.5)
Transport options:			
Access to car	44 (91.7)	224 (68.1)	195 (55.7)
Access to STS	–	42 (13.6)	68 (19.5)
Uses public transport (bus)	31 (64.7)	97 (29.4)	81 (23.3)
Dependent on walking or public transport (no car or STS)	–	60 (18.7)	93 (26.6)

*Notes*: OPUS: ‘Older People's Use of Unfamiliar Space’. STS: special transport services. 1. Based on total sample.

#### UK study

For the OPUS study a final sample of 44 volunteers 60 years and older (one person was 59 years old) was recruited from organisations for older adults (University of the Third Age, 50+ forum) in the area. In the qualitative part of the study, the group of ten older people (59–84 years old, six women and four men) who travelled to the unfamiliar town were self-selecting from the 44 (Phillips *et al.* 2013).

#### Swedish studies

The participants in the ‘Let's Go for a Walk’ study were older people (65 years and older) living in a defined study district in central Kristianstad. All of the 526 older residents received the postal questionnaires of whom 330 persons (60%) responded to the before-questionnaire in 2002 and 347 persons (66%) to the after-questionnaire in 2007; 195 persons had replied on both occasions and could therefore be included in a longitudinal study group. Participants for the following studies (research circles, participant observations and focus group interviews) were recruited among those responding positively to further project involvement.

In the qualitative parts of the ‘Let's Go for a Walk’ study, six persons (71–84 years old, four women and two men) took part in the participant observations before intervention and 11 after (67–89 years old, six women and five men). Eight persons representing older people participated in the research circles together with eight representatives from the municipality. One focus group was held after intervention with five participants (67–84 years old, two women and three men). The participants were recruited and strategically selected from the questionnaire sample as previously reported in Ståhl *et al.* (2008) and Ståhl, Horstmann and Iwarsson (2013).

The participants in the ‘Walking in Old Age’ study were older people (65 years and older) living in a defined study district in central Hässleholm, a medium-sized city in the south of Sweden. All of the 616 older residents in the study district were included in the sample of the before-questionnaire. The response rate in the before-questionnaire was 58 per cent (N = 356). For the after-questionnaire, those respondents who had filled in the before-questionnaire were included in the sample. The after-questionnaire also excluded those respondents who had died or moved from the study district during the implementation period: 332 respondents remained. The response rate in the after-questionnaire was 73 per cent (N = 244).

In the qualitative parts of the ‘Walking in Old Age’ study, respondents for the focus group interviews conducted before intervention were recruited by voluntary entry among older people visiting the local senior centre in the study district. Two focus groups were held with four people in one group (people using mobility devices) and five in another group (people not using mobility devices), in total nine people (68–93 years old, four women and five men). Another ten people participated in the focus group interviews after intervention and these participants were recruited from the questionnaire respondents. Four people took part in the participant observations before intervention (84–95 years old, all women and all used mobility devices) and three of them also after intervention. Participants for these observations were recruited among the focus group participants before intervention, as reported in Wennberg, Ståhl and Hydén (2009*a*) and Wennberg, Hydén and Ståhl (2010).

## Results on mobility barriers for older people as pedestrians

This section reports on what the methods in the OPUS study (UK) and the Swedish studies ‘Let's Go for a Walk’ and ‘Walking in Old Age’ captured in relation to the perceived barriers and fears, satisfaction with outdoor environment and walking behaviours. The results focus on reporting similarities and differences between the studies mainly based on quantitative studies, but with qualitative findings illustrated by quotations to supplement and enrich the data.

### Characteristics of the respondents

In all, 751 older people (59–99 years old) participated in the three studies conducted in two European countries, showing both differences and similarities in the characteristics (Table 3). The mean age in the two Swedish studies were 76 and 78, respectively, whilst the British OPUS study also included people under 65 and had a mean age of 71. The proportion of men and women who participated is quite similar in the three studies (around 35 per cent men and 65 per cent women) as well as the proportion of couple and single households (around 55 per cent of the respondents lived in couple households and 45 per cent in single households).

The sample in the ‘Let's Go for a Walk’ study showed more functional limitations than in the ‘Walking in Old Age’ study. There were no major differences in the reliance on mobility devices though. The OPUS study collected no data on functional limitations and mobility devices. However, the question on perceived health revealed a rather satisfied sample with respect to their health condition (58 per cent were always satisfied) with a rather high ability to carry out daily activities (73 per cent were always able to carry out daily activities).

In all three studies, a majority of the respondents had access to a car, in particular in the OPUS study where 92 per cent had this access compared with 68 and 56 per cent in the Swedish studies. Nineteen per cent of the respondents in the ‘Let's Go for a Walk’ study and 27 per cent in the ‘Walking in Old Age’ study were dependent on walking as their transport mode in the sense that these respondents had neither access to a car nor to special transport services.

A majority of the respondents take a walk several times a week (96 per cent in the OPUS study, 85 per cent in the ‘Let's Go for a Walk’ study and 61 per cent in the ‘Walking in Old Age’ study). However, there are those respondents who rarely walk outdoors, especially in the ‘Walking in Old Age’ study where 15 per cent only go out once a month or even more seldom.

### Perceived physical barriers

Despite different quantitative measures, similar findings emerged across all three studies, including perceived physical barriers (Table 4). Physical barriers such as high kerbs and holes/unevenness on pavements are pointed out by one in six older people in the samples in both the UK and Sweden. Slopes and leaning pavements cause problems, especially for people using a rollator or wheelchair. Both in the OPUS study and the ‘Walking in Old Age’ study, the respondents also mention that such barriers become even more problematic as you age:

Another thing is the state of the pavements, appalling. (Female, 69)

**Table 4.** Physical barriers when walking in the neighbourhood

	OPUS	‘Let's Go for a Walk’		‘Walking in Old Age’	
		Before	After	Before	After
N	44^1^	195^2^		244^2^	
	*Frequencies (%)*				
Poor snow removal/ice prevention	–	136 (69.7)	44 (22.6)	–	–
Ice and slipperiness	–	–	–	114 (46.7)	102 (41.8)
Poor snow removal	–	–	–	100 (41.0)	86 (35.2)
Cyclists on pavements/footpaths	–	102 (52.3)	40 (20.5)	96 (39.3)	87 (35.7)
High kerbs	–	31 (15.9)	20 (10.3)	35 (14.3)	44 (18.0)
Few benches	–	53 (29.6)	16 (8.2)	37 (15.2)	35 (14.3)
Holes and unevenness on pavements	–	33 (16.9)	9 (4.6)	41 (16.8)	44 (18.0)
Mopeds on pavements/footpaths	–	42 (21.5)	13 (6.7)	38 (15.6)	28 (11.5)
Poor lighting	7 (14.6)	12 (6.2)	5 (2.6)	23 (9.4)	19 (7.8)
High speeds	31 (64.6)	29 (14.9)	14 (7.2)	25 (10.2)	25 (10.2)
High traffic volumes	19 (39.6)	26 (13.3)	12 (6.2)	16 (6.6)	12 (4.9)
Few pedestrian crossings	19 (39.6)	–	–	9 (3.7)	7 (2.9)
Short green lights	–	40 (20.5)	19 (9.7)	11 (4.5)	10 (4.1)
Difficult to read signs	–	1 (0.5)	3 (1.5)	7 (2.9)	5 (2.0)
Slopes/hilliness	27 (56.2)	4 (2.1)	0 (0.0)	8 (3.3)	6 (2.5)
Lack of pavements	3 (6.3)	–	–	–	–
Major roads, railways, rivers, *etc*.	1 (2.1)	–	–	–	–

*Notes*: OPUS: ‘Older People's Use of Unfamiliar Space’. 1. Based on total sample. 2. Based on before/after data.

Another reported problem in the three studies is cyclists and moped riders using pavements and footpaths or disturbing and hindering features on the pavements. Parked bicycles and mopeds were emphasised as being both annoying, unsafe and hindering when getting around by many of the respondents in the two Swedish studies. In the OPUS study, similar temporal barriers were expressed by the respondents, such as parked cars on pavements, rubbish bins, advertising stands and other street furniture:

They do seem to love putting obstacles on all these pavements. (Male, 70)

It's more and more popular nowadays this parking on pavements, which I'm not too happy about especially if you are disabled, it'll cause quite a problem there, I think. (Female, 78)

Since I have started to use my rollator, I immediately noticed how high the kerbs were as well as all other types of barriers. (Female, 93)

Since I have been in a wheelchair or scooter I do find that the surfaces of the roads – High Street particularly – does go at an angle and it is not a very nice feeling when you are on a scooter as you feel you are going to tip off. (Female, 75)

### Fears

Safety and security, both related to traffic and to crime, are other issues of great concern in the three studies for older people's outdoor mobility (Table 5).

**Table 5.** Fears when walking in the neighbourhood

	OPUS	‘Let's Go for a Walk’		‘Walking in Old Age’	
		Before	After	Before	After
N	44^1^	195^2^		244^2^	
	*Frequencies (%)*				
Fear of crime (during day)	3 (6.3)	–	–	–	–
Fear of crime (during night)	9 (18.8)	–	–	–	–
Fear of crime (general)	–	40 (20.5)	16 (8.2)	97 (39.8)	97 (39.8)
Fear of falling	–	26 (13.3)	12 (6.2)	41 (16.8)	39 (16.0)
Fear of crossing the street	–	14 (7.2)	11 (5.6)	12 (4.9)	13 (5.3)
Fear of involvement in traffic accident	–	7 (3.6)	9 (4.6)	14 (5.7)	5 (2.0)
General feeling of anxiety	1 (2.1)	11 (5.6)	9 (4.6)	48 (19.7)	42 (17.2)
Fear of getting lost	3 (6.3)	–	–	–	–

*Notes*: OPUS: ‘Older People's Use of Unfamiliar Space’. 1. Based on total sample. 2. Based on before/after data.

Specific traffic-related fears are connected to cyclists and moped riders on pavements and footpaths as well as conflicts with motorised traffic when crossing the street. Around 4–5 per cent of the respondents in the two Swedish studies reported fear of being involved in a traffic accident. Likewise, anxiety when crossing the street is often mentioned due to high traffic volumes and high speeds, but also due to a lack of consideration for vulnerable road users from car drivers. One of the interviewees in the ‘Let's Go for a Walk’ study expressed:

I do not feel safe; I have to stay alert all time and be careful. Cyclists approach fast and the modern ones are really quiet. They never give any signal. I get scared when they pass by. There are more and more cyclists – and moped riders. (Female, 84)

In the OPUS study, the buses were also pointed out as a danger to pedestrians:

The buses were just horrendous and I am surprised that you have not had a lot more accidents, especially in Osborne Street where the buses pull in, because it's frightening really. (Male, 72)

### Satisfaction

Even though older people encounter several barriers when walking, a majority of the respondents in all three studies are very satisfied with the outdoor environment in their local neighbourhood (Table 6).

**Table 6.** Satisfaction with outdoor environment

	OPUS	‘Let's Go for a Walk’	‘Walking in Old Age’
N^1^	44	347	356
	*Frequencies (%)*		
Satisfaction with outdoor environment in the neighbourhood:			
Mean score	–	5.61	5.63
1. Very poor	–	4 (1.2)	6 (1.8)
2.	–	4 (1.2)	2 (0.6)
3.	–	13 (3.8)	16 (4.7)
4.	–	45 (13.2)	42 (12.3)
5.	–	77 (22.5)	72 (21.1)
6.	–	89 (26.0)	86 (25.2)
7. Excellent, could not be better	–	110 (32.2)	117 (34.3)
Satisfaction with shopping facilities in the neighbourhood:			
Never satisfied	2 (4.2)	–	–
Sometimes satisfied	14 (29.2)	–	–
Quite often satisfied	17 (35.4)	–	–
Always satisfied	15 (31.3)	–	–
Satisfaction with recreational facilities in the neighbourhood:			
Never satisfied	5 (10.4)	–	–
Sometimes satisfied	12 (25.0)	–	–
Quite often satisfied	12 (25.0)	–	–
Always satisfied	19 (39.6)	–	–

*Notes*: OPUS: ‘Older People's Use of Unfamiliar Space’. 1. Based on total sample.

The respondents in the OPUS study were also satisfied with shopping and recreational facilities in the neighbourhood. However, respondents in the ‘Walking in Old Age’ study mentioned problems with too long a walking distance to their nearest grocery store as stores had moved out from the central city district in recent years to more car-oriented locations.

The Swedish studies involved implementation of various improvements of the outdoor environment in the study districts which were generally appreciated by the respondents (Table 7). Especially more benches, lowered kerbs at pedestrian crossings as well as more even and smooth pavements were highlighted. However, problems are still to be solved in the neighbourhood according to the respondents. One respondent stated that older people still do not dare to go out due to fear of crime and then well-designed walkways are of no significance:

It is alarming that you cannot feel safe in your own neighbourhood. Then these other measures [accessibility measures] don't matter. (Male, 85)

**Table 7.** Satisfaction with implementations in the local outdoor environment

	OPUS	‘Let's Go for a Walk’	‘Walking in Old Age’
N	44^1^	195^2^	244^2^
	*Frequencies (%)*		
Satisfaction with more even and smooth pavements:			
Mean score	–	3.92	4.10
1. Not of any significance	–	6 (3.6)	4 (2.0)
2.	–	9 (5.4)	5 (2.5)
3.	–	38 (22.8)	49 (24.4)
4.	–	54 (32.3)	51 (25.4)
5. Of great significance	–	60 (35.9)	92 (45.8)
Satisfaction with lowered kerbs at pedestrians crossings:			
Mean score	–	4.02	3.99
1. Not of any significance	–	9 (5.4)	7 (3.5)
2.	–	10 (6.0)	5 (2.5)
3.	–	24 (14.4)	54 (27.1)
4.	–	49 (29.3)	49 (24.6)
5. Of great significance	–	75 (44.9)	84 (42.2)
Satisfaction with more benches:			
Mean score	–	3.53	3.86
1. Not of any significance	–	13 (8.3)	6 (3.1)
2.	–	15 (9.6)	16 (8.2)
3.	–	48 (30.6)	51 (26.2)
4.	–	38 (24.2)	49 (25.1)
5. Of great significance	–	43 (27.4)	73 (37.4)
Satisfaction with moved and improved bus stops:			
Mean score	–	2.34	4.34
1. Not of any significance	–	56 (43.4)	5 (2.5)
2.	–	19 (14.7)	6 (3.0)
3.	–	26 (20.2)	26 (13.1)
4.	–	10 (7.8)	41 (20.7)
5. Of great significance	–	18 (14.0)	120 (60.6)
Satisfaction with measures in general:			
Mean score	–	3.76	4.01
1. Not of any significance	–	9 (5.0)	5 (2.6)
2.	–	12 (6.6)	6 (3.1)
3.	–	46 (25.4)	47 (24.6)
4.	–	61 (33.7)	57 (29.8)
5. Of great significance	–	53 (29.3)	76 (39.8)
Satisfaction with changes in the local area in recent years:			
Never satisfied	1 (2.1)	–	–
Sometimes satisfied	16 (33.3)	–	–
Quite often satisfied	25 (52.1)	–	–
Always satisfied	6 (12.5)	–	–

*Notes*: OPUS: ‘Older People's Use of Unfamiliar Space’. 1. Based on total sample. 2. Based on after data.

## Discussion and comments

This paper has re-analysed and compared data from three studies from different locales in two countries (UK and Sweden) to provide a comprehensive account of the issues facing older people in their outdoor environments. In this section, we evaluate the comparative approach of this paper and discuss transferability of research questions, methods and findings between the two countries (and the three studies). Advantages and success factors of the comparative approach are discussed, as well as the considerations.

### Advantages and success factors of a cross-country comparative approach

Providing methodological verification or triangulation of a particular phenomenon or theme is a benefit of cross-national comparative analysis (Denzin 1970; Hantrais 2008). The comparative analysis in this paper shows similar findings concerning the barriers and fears as perceived by older people in the UK study and in the two Swedish studies which strengthens the findings. Using such data to triangulate or verify concepts and themes proved useful, reinforcing the key issues that older people perceive as important in their environment. All the studies engaged older people with similar demographic characteristics – many were active within their community and walked regularly; consequently, they were well placed to discuss pedestrian barriers, fears and other issues. Key issues were also reinforced by each focus group of older people and obstructions on pavements such as bicycles, parked cars or street furniture were reasons for avoiding areas. High kerbs, slopes and leaning pavements were also a barrier across all sites. In relation to fears, safety and security around traffic and crime were global concerns, along with high traffic volumes and speeds and lack of consideration of car drivers, cyclists and moped users. Thus, findings in one study can be verified in another and add weight to the need to develop strategies to improve the built environment.

There were a number of similarities and common ground in the three studies that provided a great advantage in comparing data. These included the context of the built environment, the conceptual base, a mixed-method approach and participation of older people in the research. The environment studied in both countries was similar, with a focus on the *built urban environment* as opposed to the rural natural environment, as well as on the *public outdoor environment* as opposed to private outdoor environments or indoor environments. The OPUS study included two large-scale towns: one familiar to older people in Wales and one unfamiliar in England. The Swedish studies concentrated on two medium-sized cities in the south of Sweden in which a study district was defined in the central district of the cities where many older people live. Additionally, within the urban environment there was utility in all studies in looking at pedestrian routes rather than physical features or origins and destinations in the environment, as a route has to be accessible and safe to older people. This highlights the importance of contextualising the pedestrian experience in the broadest environmental context as possible and looking at the *‘travel chain’ perspective* (Ståhl 1997). Design of individual buildings and pavements is important but it is the route that people follow that provides the whole pedestrian experience.

All three studies draw on the Person–Environment fit (Lawton and Nahemow 1973) and the related Person–Environment–Activity fit (Iwarsson and Ståhl 2003) as a guiding conceptual base. This common conceptual base allowed us to test theory against practice and to explore the utility of this concept across different geographical contexts. Given the issues raised by older people in all three studies, the Person–Environment–Activity fit has utility as there are improvements in the environment which can be made to improve wellbeing. Additionally, the authors coming from different disciplinary backgrounds but with gerontology being the focus of the study were able to link disparate disciplines together – transport and traffic studies with sociological and geographical literatures. This added to the conceptual framework of the study and challenged the dominant stance of the Person–Environment framework which has a problem-facing approach. It ensured that activity and agency were major considerations in the framework.

*Mixed methods* were used in all three studies. Exploring older people's perceptions as pedestrians as well as objective measures in the environment added rich narratives to the study. The OPUS study combined both objective data of the environment (through the UDQ) with the narratives of older people from the cave and the walk around town. The OPUS study used a concurrent nested design with the integration of mixed methods coming at the data collection phase. In relation to the qualitative data, grounded theory and narrative approaches were used to analyse the data. This enabled us to gain a much broader perspective with the survey as a background to the qualitative data.

Similarly, the Swedish studies used focus group interviews and participant observations with the critical incident technique (Flanagan 1954; Jensen, Iwarsson and Ståhl 2002) to supplement postal questionnaires. Although the methods used to explore the objective assessment of the environment were different in each study, the combination of objective and subjective, mixed-method data is important in attaining a complete picture of the urban environment, *i.e.* triangulation. The two Swedish studies were before–after studies involving intervention and the qualitative methods were also used to explain the quantitative evaluation of implemented measures in the local neighbourhood, *i.e.* an explanatory mixed-method design (Creswell and Plano Clark 2007). Qualitative themes collected through similar means, such as the focus groups and narratives (OPUS) as well as the participant observations with the critical incident technique and research circles, consequently reflected the etic dimensions of the experiences of older people when confronted by similar urban experiences.

All three studies applied *participatory research*, highlighting the importance of the voices of older people and involving older people in research (Kylberg *et al.* 2017). Older people were involved not only as sources of *data* but also as *partners* (Reed, Weiner and Cook 2004; Reed *et al.* 2008). This was particularly prevalent in the ‘Let's Go for a Walk’ study which included older people in the whole process from describing barriers in their local outdoor environment to suggesting, prioritising and deciding on measures to eliminate these barriers. A significant factor in older people's use of the outdoor environment (familiar or unfamiliar) is how they perceived that environment. All studies found that safety and aesthetics were important in conveying meaning in the environment; captured through older people's voices. The methodology employed in all studies was able to demonstrate these issues because it used objective measures (*e.g.* SWEAT-R and UDQ in the OPUS study) in combination with older people's voices. Objective measures of the environment can be enhanced by a sense of meaning and history important for older people.

### Considerations when comparing data and drawing conclusions

An obvious challenge of conducting secondary research of this nature is that there are missing comparable data as well as different response rates and scales. The use of existing objective assessment methods (*e.g.* SWEAT-R and UDQ were applied in the OPUS study) when applicable or well-recognised approaches when formulating questions in study-specific questionnaires (*e.g.* the Housing Enabler concept and a global question on perceived health by Tibblin *et al.* (1990) were applied in both Swedish studies) facilitated valid and comparable findings. Both studies also applied similar five-point Likert scales which also ensured comparability, even though response rates and alternatives (*e.g.* 1 = very unimportant, 2 = important, 3 = neither, 4 = important, 5 = very important) differed to some extent. Different response rates and alternatives could be handled either by slightly adjusting the response alternatives of a study to the prevailing ones (if the difference was small) or by reporting studies separately. For example, all three studies examined older people's ability to walk a certain distance, but the OPUS question had a completely different approach than the similar question in ‘Let's Go for a Walk’ and ‘Walking in Old Age’ (*see* Table 3) and had to be kept separate.

Making comparisons between countries based on data collected in different ways, as above, poses challenges in terms of data harmonisation. There are no well-established standard procedures for retrospective data harmonisation, particularly without similar protocols, questions or databases. However, although the methods of the studies were different, the information generated similar meaning to allow the studies to be pooled. Further analysis suggested that findings from the critical incident technique paralleled those of the UDQ, with respondents choosing the area on which to comment; guided walks were similar in both countries. The research circles and focus groups with planners were similar in terms of how they were conducted and with whom.

There were a number of methods that were unique to each study which did not allow for comparison. In the OPUS study, older people's experiences of an environment in a reality cave cannot necessarily be matched by what they experience in reality. Fears around the business of traffic, noise and sensory overload came from the walk around town rather than the narratives shared within the cave setting. The Swedish studies looked at legislation and policy as well as perceptions before and after intervention, which was not undertaken in the UK study. Despite such differences it is important to reflect on the findings within each country context and to transfer lessons that could be applied from one study to the other.

In any cross-national study attention needs to be paid to the generalisation from a small-scale study to national generalisation. Similarly, there are several different socio-cultural differences which cannot be easily transferred from one country to another. The paper considers data across two similar European countries; comparison with other countries with differing socio-cultural differences may render the analysis ineffective and caution needs to be taken in the assumptions of transferability. Some differences were observed between countries as described below.

The age definition differed between the studies, where the UK study included people 60 years and older and the two Swedish studies included people 65 years and older. Older people were in general more fit in the OPUS study than in the Swedish studies, which can be explained partly by the different age definition, where health status and functional capacity decline with increasing age (Löfqvist *et al.* 2007; Parker *et al.* 2008).

All three studies are likely suffering from the common sampling bias as other similar studies in the field of older people's perception – that is the under-representation of very old people since larger proportions of them live in residential establishments (Gubrium and Holstein 2001). The mixed-method approach and the strategic sampling strategies applied in the three studies reported in this paper facilitate inclusion of the voices of very old people and of older people with different functional limitation.

There are also country-specific preconditions affecting the design of the questionnaires and other methods in the studies and thereby the comparability. Sweden faces other accessibility challenges during winter related to snow and ice. Therefore, these aspects were included in the Swedish studies and were rated very high by older people whilst not mentioned in the UK study. Furthermore, the accessibility legislation in Sweden which was focused on in the interventions of both Swedish studies contributed to putting ‘access for all’ high on the agenda in local and regional authorities at the beginning of the 21st century. The disability organisations have been quite successful in communicating their visions and ambitions to society (including older people). Hence, older people most likely have some expectations regarding the design of their local neighbourhood and on their possibilities of getting around in the city and region with public transport, which affect their response. These expectations will likely be further reinforced in studies where the local and regional authorities participate. When drawing conclusions from the Swedish studies given the Swedish context, accessibility issues are more likely to be higher rated than in the UK study.

## Conclusions, methodological recommendations and implications for planning

This paper highlights that you can compare cross-nationally using a mixture of similar and different methodology and that there are lessons that are transferable. The comparative analysis in this paper was facilitated by the similarities and common ground in the three studies, including the context of the built environment, the conceptual base, a mixed-method approach and participation of older people in the research. Based on these success factors, the following methodological recommendation for future studies on older people's perceptions as pedestrians can be formulated:

• Methods and findings should be related to prevailing theoretical frameworks in order to verify (or challenge) the common ground, *e.g.* the Person–Environment fit (Lawton and Nahemow 1973) and the related Person–Environment–Activity fit (Iwarsson and Ståhl 2003), as in this paper.
• Existing objective assessment methods should be applied when applicable (*e.g.* SWEAT-R and UDQ were applied in the OPUS study) or well-recognised approaches when formulating questions in study-specific questionnaires (*e.g.* the Housing Enabler concept and a global question on perceived health by Tibblin *et al.* (1990) in the Swedish studies) ensures valid and comparable findings.
• Study constraints should be carefully defined and described in order to ensure comparative analysis, *e.g.* age definitions, characteristics of respondents, inclusion criteria in sampling, *etc*. It is recommended to use common age definitions, *e.g.* older people as 65 + . Be aware that standardised national data-sets, *e.g*. national travel surveys, often excluded people 75 years and older.
• Ensure representative sampling of older people in order to get a complete picture of needs and preferences, *e.g.* choose an inclusive sampling strategy (*e.g.* very old people and people not living in their home are hard to reach) and inclusive research methods (*e.g.* traditional postal questionnaires are likely not suitable for people with visual and cognitive functional limitations).
• A mixed-method approach is recommended when examining a broad question such as older people's perceptions as pedestrians, involving several research disciplines. Emic and etic perspectives need to be considered.
• Involve the voice of older people in research and planning, both as source of data and as partner, in order to get a complete and correct picture of their needs and preferences. In all three studies, they were involved in the whole process from describing barriers in the neighbourhood, suggesting measures to eliminate these barriers, and prioritising and deciding on measures to be taken in co-operation with stakeholders and decision makers. User involvement leads to research of greater relevance to people and the findings are more likely to be implemented, and not least, such research might also foster empowerment of the public (Fudge, Wolfe and McKevitt 2007; Kylberg *et al.* 2017).
• Be aware of social, historical and cultural differences between countries – knowledge on such country-specific preconditions are needed in order to draw valid conclusions from data collected in different countries.
• Methodology has to be sensitive to the local context and this should be recognised in any analysis.

Comparative analyses of diverse data-sets, as we have illustrated in this paper, can identify the key issues and reinforce findings across different contexts. Through our comparable findings we are able to describe the key ingredients of a safe environment in both countries and reflect on findings from one country in light of another country. Both the UK and Swedish studies highlighted the importance of the immediate locality and neighbourhood to people in old age and the need for more-accessible neighbourhoods to enable people to go out more. Locality remains an important spatial reference in later life. This demonstrates the importance of looking to existing data-sets to ‘add value’ in cross-national analysis.

All three studies highlighted that, even with improvements in terms of barrier-free design, if people are fearful of traffic and crime then they will not go out. Key ingredients of a safe environment include the inclusive design of the built environment and simple measures for improvement, *e.g.* low kerbs and more benches. Reducing traffic volumes and speeds where there are pedestrians and clear separation of vulnerable road users (pedestrians, cyclists and mopeds) are safety/security-related issues that have also been emphasised by other studies (*cf*. Mollenkopf *et al.* 2004*b*; Risser, Haindl and Ståhl 2010).

One of the key findings was the satisfaction that older people expressed with their local environment. This is important as the radius of movement of older people may shrink with age, making the local neighbourhood even more important to them. The wider context of environmental planning is therefore crucial in decisions about where to locate commercial centres – many out-of-town centres impact on older people in negative ways, relinquishing their opportunity for social contact as well as their independence to walk within their familiar environment to shops and other facilities. A car-oriented approach in planning is counteracting the needs of the ageing population where walking and public transport are the predominant transport modes.

Accessibility for older people as pedestrians concerns barrier-free design of outdoor environments as well as ensuring access to desired places and activities within walking distance. Furthermore, a *year-round perspective* on accessibility issues should be considered in several countries, *e.g.* in Sweden, where climate-responsive design and winter maintenance strategies are important in order to ensure pedestrian accessibility all the year round.

The challenges and issues facing older people as pedestrians have been put higher on the agenda at both the policy and research arena on European, national and local levels. However, in actual planning there is still much to be accomplished in order to facilitate independent mobility in old age. The implications of an ageing population are not factored in routinely to the planning system in the UK and Sweden (Hallgrimsdottir *et al.* 2016). Where there is discussion (as in the OPUS focus group), it is in relation to housing, mobility and accessibility, and less on the impact of urban design on older people (Hockey, Phillips and Walford 2013). The planners in the OPUS focus group still focused on problem-based issues around housing and accessibility and less on the link between wellbeing and the environment. Promoting socially inclusive communities and neighbourhoods is a requirement of the planning process (Department of Communities and Local Government 2007) yet there is variability across the UK and little practice based on the voice of local older people. Planners did not factor in the cultural and social barriers that people faced, but concentrated on the physical issues that could be remedied. In Sweden, the accessibility legislation has contributed to putting ‘access for all’ on the agenda of local and regional authorities (and for private property owners) during the 2000s, but still there is a lack of awareness and knowledge regarding the needs of people with different functional limitations (including older people) and ‘design for all’ concepts among all factors involved in the planning process, and there is also a large variation between authorities across Sweden (Hallgrimsdottir *et al.* 2016; Wennberg 2011; Wennberg, Ståhl and Hydén 2009*b*). Planners are aware of the cultural and social barriers that older people face, but much more needs to be done concretely and in such processes the voices of older people need to be more integrated.

In conclusion, given the increasing diminution of research resources for cross-cultural studies it is important to re-analyse secondary data and compare studies which coalesce around a particular theme. Comparing methods used to assess their comparability in findings and to draw on similar methodologies to provide solutions will be of increasing importance. The paper compares two similar socio-cultural locales in two different countries to demonstrate how this could be taken forward. It highlights the benefits of retrospectively comparing countries using similar methods and conceptual frameworks to add to our understanding of issues facing older people in the outdoor environment.
